# Connectivity adaptations in dopaminergic systems define the brain maturity of investors

**DOI:** 10.1038/s41598-021-91227-x

**Published:** 2021-06-03

**Authors:** Elena Ortiz-Teran, Ibai Diez, Jorge Sepulcre, Joaquin Lopez-Pascual, Tomas Ortiz

**Affiliations:** 1 Gordon Center for Medical Imaging, Department of Radiology, Massachusetts General Hospital, Harvard Medical School, Boston, MA 02115 USA; 2 Facultad de Ciencias Jurídicas y Sociales, Universidad Rey Juan Carlos, 28032 Madrid, Spain; 3 Departamento de Economía de la Empresa, Facultad de Ciencias Jurídicas y Sociales, Universidad Rey Juan Carlos, 28032 Madrid, Spain; 4 Departamento de Medicina Legal, Psiquiatría y Patología, Facultad de Medicina, Universidad Complutense de Madrid, 28040 Madrid, Spain

**Keywords:** Reward, Synaptic plasticity

## Abstract

Investment decisions rely on perceptions from external stimuli along with the integration of inner brain-body signals, all of which are shaped by experience. As experience is capable of molding both the structure and function of the human brain, we have used a novel neuroimaging connectomic-genetic approach to investigate the influence of investment work experience on brain anatomy. We found that senior investors display higher gray matter volume and increased structural brain connectivity in dopamine-related pathways, as well as a set of genes functionally associated with adrenaline and noradrenaline biosynthesis (SLC6A3, TH and SLC18A2), which is seemingly involved in reward processing and bodily stress responses during financial trading. These results suggest the key role of catecholamines in the way senior investors harness their emotions while raising bodily awareness as they grow in investment maturity.

## Introduction

Humans have a tendency to predict future events not only in foreseeable scenarios, but also in the midst of randomness. Obviously, not all predictions are accurate, especially in the financial markets. It is sometimes claimed that the stock market is a game of chance, where no amount of knowledge of past performance data can help to predict future market trends^1^. However, instead of seeing random variability in market movements, our brain is designed to impose a pattern^2^ in pursuit of allocating money advantageously. In this context, neuroscientists have found that investment decisions may be driven by signals triggered in dopamine-rich subcortical areas of the brain^3^, where more unconscious processes are expected to take place.

It has been postulated that dopamine neurons help to predict rewards by detecting subtle patterns that we would otherwise not immediately comprehend. However, this perception only comes from experience, by a learning process of constantly readjusting expectations based on actual results^4^. Once these predictions are converted into internal feelings, perceived somatic signals appear in anticipation of those expected outcomes^5,6^. It is probable that this nonconscious process guides our decisions prior to our conscious knowledge and provides the neurobiological evidence as to why these choices are made as they “feel right” or come “straight from the gut”^5^, even with high-stakes decisions^7^.

Notwithstanding that interoception may influence cognitive and affective processes involved in risky decision-making^8^, individuals differ in their ability to generate and sense physiological responses to emotional situations^9^. Experienced traders, for instance, have lower heart rate variability^10^, but also a better acuteness for discerning their own heartbeat, a sensitivity that has been connected to their profitability as well as their survival in the financial markets^11^. Considering their connection to the dopaminergic reward system, it is not surprising that the brain areas involved in what has been termed “brain-gut” neural communication^12,13^ happen to be what the literature is highlighting as some of the key regions responsible for risk taking in economic investments^14^.

In an attempt to reveal the success of the intelligent investor, neuroeconomics has begun to connect brain areas supporting this decision-making process to real-life financial risk taking. Thus far, only the activation in the anterior insula^15^ and the ventrolateral prefrontal cortex^16^ have been negatively correlated with individuals’ expertise in trading stocks in real life. Furthermore, the decision to trade in active investors has recently been attributed to genes associated with catecholamine synaptic levels^17^, especially dopamine^18,19^ since is closely connected to reward-seeking behaviors^20^. Although economic preferences are partially explained by genetic differences^21^, environmental factors, such as work experience, also mediate in this relationship between genes and risk-taking^18^ shaping individuals’ financial decisions and may even diminish genetic predispositions to investment biases^22^.

In light of these findings, it can be hypothesized that the experienced investor unwittingly relies on the emotions or inner feelings induced by dopamine neurons while creating an investment portfolio. As the human brain is plastic throughout the entire lifespan^23,24^, substantial structural changes can be found in all human beings with particular behavioral expertise^25–27^. This study investigated whether work experience investing in the financial markets could lead to brain network changes, especially in dopamine-related systems, using a neuroimaging connectomic-genetic integration approach.

## Results

### Investment experience and brain changes

**Figure 1:**
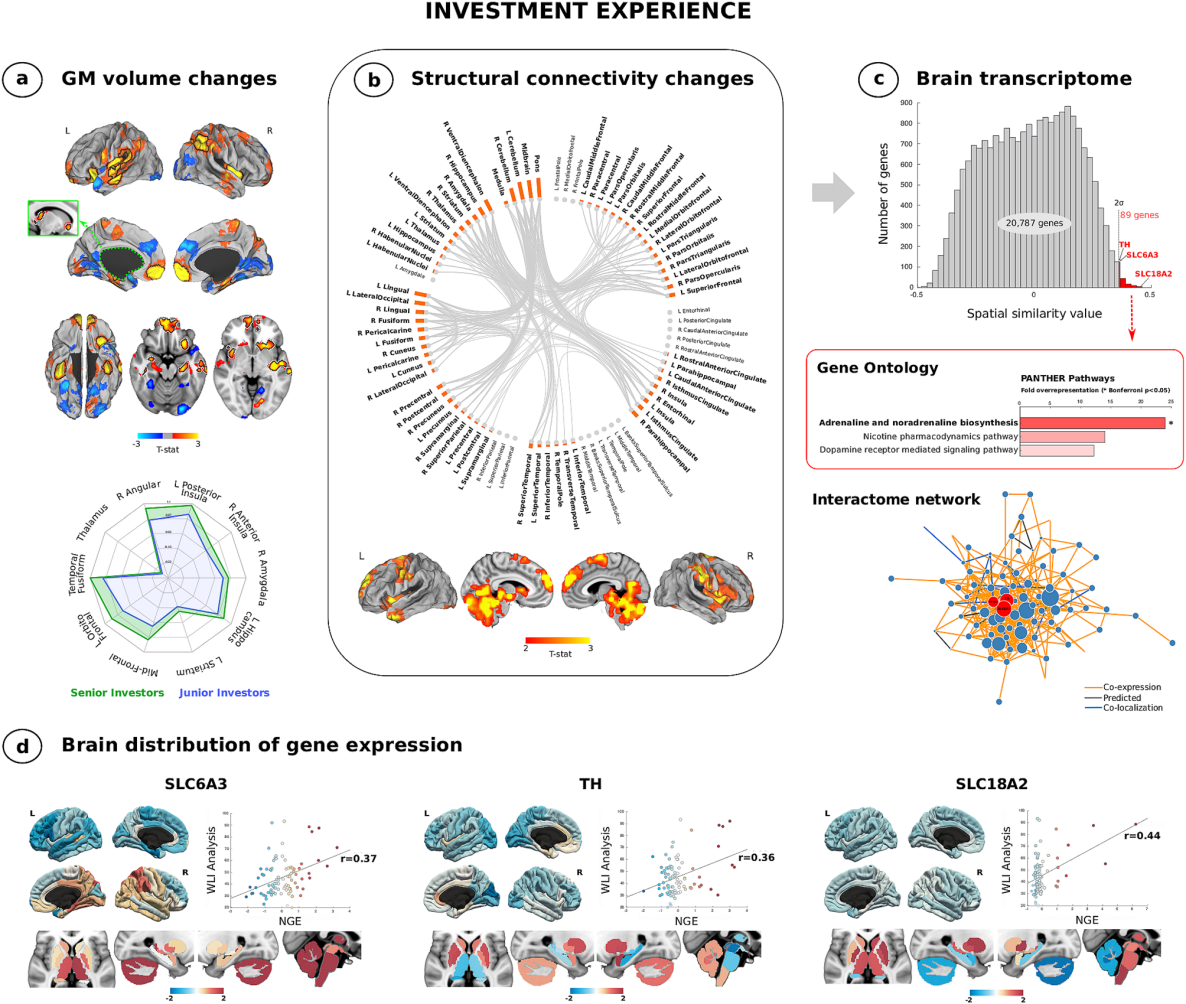
Structural brain changes and cerebral gene expression related to investment experience. (**a**) Cortical and subcortical maps of gray matter volume in senior investors. Red-yellow colors represent higher volume and blue colors lower volume. The results corrected for multiple comparisons are outlined in black (*Top*). Radar plot is shown (*Bottom*). (**b**) Connectogram of structural connectivity and brain projections of the weighted-degree of link-level interaction analysis related investment work experience. (**c**) Genes whose similar spatial distribution correlated with brain connectivity maps shown in b (*top*). Genetic pathways associated with these 89 genes (*middle*). Genetic functional network exhibiting the centrality of SLC6A3, TH and SLC18A2 (*bottom*). (**d**) Projections of cortical and subcortical areas of the distribution of these genes in the brain. SLC6A3 (p = 0.00029557), TH (p = 0.00043982) and SLC18A2 (p = 0.00001728). *WLI analysis* weighted-degree of link-level interaction analysis, *NGE* normalized gene expression, *R* right, *L* left.

We found that senior investors display higher gray matter (GM) volume in several brain areas compared to junior investors, namely in the bilateral medial prefrontal cortex, frontal pole, insular cortex, thalamus, fusiform cortex and inferior temporal gyrus; right amygdala, putamen, angular gyrus, superior parietal lobule and lateral occipital cortex; and left orbitofrontal cortex, ventral striatum, hippocampus, superior and middle temporal gyrus (Fig. 1a and Table S1). No regions showed significant decreased volume after carrying out the same analysis. Interaction analysis showed increased structural brain connectivity in the mesolimbic circuits, the cerebellum, the insular cortex and the temporal pole when comparing groups (Fig. S1). When we gathered all the participants in one group and took into consideration work experience, we observed a predominant pattern of subcortical and mesocortical connectivity, particularly involving the brainstem, such as the pons and the midbrain (Fig. 1b). All the statistical and significant link-level connections for both analyses can be found in the connectogram figures (Fig. 1b and Fig. S1).

### Intersection between investor brain connectivity and cerebral gene expression

While evaluating the entire cerebral transcriptome, we discovered that the cortical expression levels of 89 genes showed significant spatial similarity with the brain connectivity associated with work experience (Fig. 1c). We identified that this set of genes is functionally related with specific genetic pathways, such as the adrenaline and noradrenaline biosynthesis, the nicotine pharmacodynamics pathway and the dopamine receptor mediated signaling pathway. Using a lenient significant threshold in overrepresentation analysis showed that after applying a Bonferroni correction (p < 0.05), the adrenaline and noradrenaline biosynthesis pathway survived (Fig. 1c). This catecholaminergic-related molecular pathway encompassed three specific genes, namely SLC6A3, TH and SLC18A2. The high spatial similarity of subcortical, and to a much lesser extent, cortical, expressions levels of SLC6A3 (r = 0.37), TH (r = 0.36) and SLC18A2 (r = 0.44) with our weighted-degree map of work experience investing in the financial markets map is displayed in surface, volume and scatterplot formats in Fig. 1d. Finally, we confirmed with an interactome genetic analysis that all three genes, SLC6A3, TH and SLC18A2, play key and interdependent roles in their overall genetic interactions beyond their mere spatial matching, based on their central position in the interactome network (Fig. 1c).

## Discussion

We found a concurrent increase in the volume of certain cortical (insula, prefrontal, temporal and parietal cortices) and subcortical areas (pons, midbrain, amygdala, hippocampus, striatum and thalamus) with regard to financial experience, which leads us to believe that none of these regions in isolation are specialized in responding to investments alone. Conversely, these results demonstrated the existence of a network that is probably engaged while considering whether to invest or not, particularly the brainstem related circuitry in the pons and midbrain. The higher genetic expression of SLC6A3, TH, and SLC18A2 in these areas suggest the importance of noradrenaline, adrenaline and dopamine, also known as catecholamines, in reward processing and bodily stress responses while trading in financial markets.

Legendary investor Peter Lynch pointed out that “*In the stock market, the most important organ is the stomach. It*’*s not the brain*”^28^, although it may be the connection between them what drives this decision-making based on “gut feelings.” The bidirectional communication system that closely connects the gut and the brain influences high and low-level processes^29^. In intuitive decision making, triggering interoceptive awareness of these signals as gut feelings is connected to fronto-insular activation^30^ when it comes to perceived risks in decision-making^31^. On the one hand, the orbitofrontal cortex seems to be one of the brain regions representing predictive reward value^32^ during economic choice^33^, whereas the ventromedial prefrontal cortex also depicts inflated trading values serving as a predictor of the tendency to ride bubbles^34^. On the other hand, the anterior insula appears to code a risk detection signal^35^ whose activation precedes riskless choices^36^, particularly in active stock traders^15^. As a consequence of experiencing these gut sensations, interoceptive memories appear within a network connecting the medial prefrontal cortex, the anterior insula, the amygdala and the hippocampus^30^; brain areas where an increased volume was seen in senior investors (Fig. 1). Paying attention to somatic signals to ensure advantageous behavior^37^ may be an ability developed by senior investors while building their portfolio.

Forecasting where the market is heading to make the most profitable investment decisions requires weighing two concepts widely used in finance: expected reward and risk. Despite the fact that reward processing involves the mesocorticolimbic dopaminergic circuit^38^, which we also observed in our results (Fig. 1), trying to calculate these parameters in a changing and uncertain environment demands a trade-off between exploitation and exploration strategies^39^. When senior investors are sure about which investment path they should follow, responding to the current conditions usually implicates exploitative decision making, where the striatum and the ventromedial prefrontal cortex track the subjective value of potential rewards^40,41^. On the contrary, seeking alternative investments or acquiring new market knowledge in order to decide may be related to exploratory decisions which are mainly associated with activation in the frontopolar cortex and the intraparietal sulcus^41^, raising the possibility that attentional control in the anterior part of the former region may be the switching mechanism among these learning behavioral plans^42^. The role of catecholamines in this regulation^43^, probably by amplifying the neural response during reward anticipation^44^ as a novelty seeking risk attitude^45^, may be one of the reasons for investors’ ability to adapt to fast-moving financial markets.

Investment decisions appear to be influenced by internal bodily states, expected outcome values and attentional mechanisms shaped by professional financial experience. Certain genes regulate the neurobiological system behind these functions, where individual differences in the neurotransmitter process may arise, impacting trading behavior^17,46,47^. By combining neuroimaging with genetic information, we were able to identify three highly expressed genes (SLC6A3, TH, and SLC18A2) involved in the regulation of noradrenaline and adrenaline, and dopamine release, previously linked to reward sensitivity, economic risk attitudes^44,45^ and exploration strategies^41^. Although prior studies have also targeted DRD4, COMT, MAOA-L and 5-HTTLPR as genetic determinants of financial risk-taking behavior, most of these results suggest that one factor mediating the variability in financial decisions is dopamine^18,48–50^, with an intermediate synaptic level associated with successful trading^17^. A feasible explanation is that by means of reinforcement learning, dopamine neurons are able to convey both positive and negative motivational signals^51^ as a probability distribution in which numerous future outcomes can be represented simultaneously in the brain^52^.

It is important to mention that our results come from a small sample size. By using neuroimaging genetics, another limitation arose from the comparison between our connectivity maps with the gene expression profiles from the Allen Human Brain Atlas (AHBA), although this represents a new approach into how environmental factors can mediate the relationship between genes with financial risk-taking in neuroeconomics. Ideally, having longitudinal MRI data could give us a more precise idea of the progression of the structural changes that occur in the brain as the years of experience increase, and whether the high rate of investors quitting their job in the early years of their career is due to factors such as a failure to develop the key circuits involved in investment decision-making. The last limitation come from the lack of longitudinal MRI data due to the high rate of investors quitting their job in the early years of their career.

Despite the fact that investors may vary in their ability to make advantageous financial choices, our conclusion is that there is a common neurological basis gained by experience. By learning how to harness the emotional aspects of investing while raising bodily awareness, they are able to turn feelings into lessons that ultimately will guide their decisions supported by catecholamine-related brain systems.

## Methods

### Participants

Thirty-one healthy participants, 16 senior investors (2 women, mean age = 40.5, SD = 7.8) and 15 junior investors (1 women, mean age = 26.5, SD = 4.3), were studied. We defined a “senior”/“junior” investor as a person who has more/less than three years of professional experience investing in financial markets according to the professional categories generally used across financial companies. All participants were recruited by the Chartered Financial Analyst Institute (CFA, Spain), the Instituto de Estudios Bursátiles (IEB, Spain) and via email through contacts. No history of neurological illness, psychiatric disorders, or substance abuse were reported. The study was conducted with the approval of the Ethics Committee of Rey Juan Carlos University and all participants provided written informed consent and were in full compliance with the Declaration of Helsinki.

### MRI data acquisition

Participants were scanned with a Siemens 3 T Magneton Prisma. To study volumetric changes in the brain, a high-resolution, 3D, T1-weighted magnetization prepared rapid gradient-echo (MPRAGE) sequence was used with: 1 mm isotropic voxels; 192 sagittal slices; acquisition matrix size = 256 × 256; repetition time (TR) = 2300 ms; echo time (TE) = 2.98 ms; field of view (FOV) = 256 mm. Participants were instructed to remain as still as possible and bi-temporal foam pads were used to restrict head motion.

### Imaging preprocessing

MRI data was preprocessed using FMRIB Software Library v5.0.7 (FSL). The anatomical T1 preprocessing pipeline included: reorientation to right-posterior-inferior (RPI); alignment to anterior and posterior commissures; skull stripping; and gray matter (GM) segmentation. An optimized Voxel-based morphometry was used^53^, carried out with FSL^54^. The partial GM volume estimations were transformed to 2 mm MNI 152 standard space using non-linear registration. The resulting images were averaged and flipped along the x-axis to create a left–right symmetric, study-specific gray matter template. Then, all native gray matter images were non-linearly registered to this study-specific template and “modulated” to correct for local expansion (or contraction) due to the non-linear component of the spatial transformation. Finally, the modulated gray matter images were smoothed with an isotropic Gaussian kernel with a sigma of 3 mm (7 mm Full width at half maximum).

### Between group structural differences

A two-class generalized linear model, adjusting for age, was used to examine between-group differences in GM volume. The removed effect of age in the brain can be visualized in Figure S2. To correct for multiple comparison, a Monte Carlo simulation was used with 50,000 iterations to estimate the probability of false positive cluster sizes at each voxel with a p-value < 0.05. The analysis was repeated correcting by gender and the results remained.

### Brain connectivity analysis

To identify possible brain networks related to GM volume changes, a structural connectivity analysis was implemented. To reduce the dimension of our link-level analysis, GM images were down-sampled to 6 mm. A general linear model (GLM), was used to examine the between group differences in the structural connectivity (the relationship of the GM volume changes in 2 regions), using an interaction analysis for each pair of GM voxels. A statistical network of 6220 × 6220 nodes was obtained. Whole-brain correction for multiple comparisons was computed adapting the Monte Carlo simulation method to networks^55^. To compute false positive cluster size with a p-value < 0.001, 10,000 random networks were generated with the same smoothing properties. Compared to weighted-degree maps where clusters are defined as contiguous voxels, here clusters were defined as links that connect contiguous voxel groups.

To study how work experience affect the different networks of the brain, another interaction analysis was used to examine the relationship of work experience with the structural connectivity strength (how strongly 2 regions’ volumes are related between them). The interaction analysis looks for multiplicative effect of the volume of one region with work experience and how this explains the volume in another region, removing the individual effects of the volume of these regions and the work experience, using a whole-brain correction for multiple comparisons computed by adapting the Monte Carlo simulation method to networks.

### Gene expression relationship with network changes

To investigate similarities between protein-coding genetic profiles with neuroimaging phenotypes, we correlated microarray gene expression data from the Allen Human Brain Atlas (AHBA) with the weighted degrees of the links with a p-value < 0.001, while performing the interaction analysis with work experience. The AHBA provides high-resolution genome-wide expression values for six human subjects, quantifying more than 20,000 genes in 3702 samples spatially distributed throughout the brain^56^. Consistent with the latest recommendations^57^, brain maps representing the spatial distribution of each gene were created by using the microarray expression data with their MRI images and the coordinates of each sample for the six donors. The following steps were performed: (i) for each gene, expression values with multiple probes were averaged; (ii) each sample was associated with an anatomical label using the 89 brain regions defined with the Freesurfer atlas (68 cortical regions defined by the Desikan–Killiany atlas, 16 subcortical regions of the Freesurfer segmentation, 2 cerebellum and 3 ROIs of the brainstem: midbrain, pons and medulla); (iii) for each subject, we computed the median of gene expressions of all the samples within the same region; (iv) finally, we computed the median gene expression value of each brain area between the six donors. After extracting the work experience weighted degree map, this was converted to the 89-region atlas and a Pearson correlation was used to assess the spatial similarity value with the expression values of all the available genes. The spatial similarity computation identified the most similarly distributed genes to our neuroimaging outcome based on weighted degree connectivity maps, using a spatial similarity value higher than 2 standard deviations of the spatial similarity distribution.

### Gene ontology (GO)

To study the genetic functionalities of genes with a highly similar spatial expression to our brain phenotype, we used Gene Ontology annotation resources. This overrepresentation test evaluated the Protein Analysis Through Evolutionary Relationships (PANTHER) pathways associated with the genes located in the upper bound of the tail. The list of genes was entered into a GO term enrichment analysis tool^58^ (http://geneontology.org), using a binomial test with False Discovery Rate correction to perform the statistical testing (multiple comparison correction at q < 0.05 level).

### Interactome analysis

We used a genetic interactome approach to validate our genetic findings beyond their spatial brain colocalizations. Genemania^59^ (http://genemania.org) and Cytoscape^60^ v3.8.2. (https://cytoscape.org) software was used to perform a genetic interactome analysis. A composite gene–gene network based in co-expressions, co-localizations, and predicted interactions was used to obtain the complex relationships between all genes derived from the neuroimaging-AHBA spatial intersections, and closeness centrality was used to assess the hubness of specific genes in the interactome network^59,60^.

### Visualization

Cortical surfaces were visualized using the population-average landmark and surface-based projections of CARET v5.65 software (http://brainvis.wustl.edu/wiki/index.php), whereas subcortical surfaces were visualized using Matlab R2018b (https://www.mathworks.com/products/matlab.html). Surface images were displayed using a color scale based on T-scores. In-volume images were added to show subcortical striatal-thalamic-limbic-midbrain findings, when present. The statistically significant links in the 6220 × 6220 matrix were projected onto a connectogram using NeuroMArVL (https://immersive.erc.monash.edu/neuromarvl/). For visualization purposes, the 6220 nodes were reduced to 85 cortical-subcortical brain regions.

## Data and code availability

Neuroimaging data and code availability: The neuroimaging dataset and all codes for imaging analysis are available for the research community from the corresponding author upon request. Genetic data: The genetic data are available from the AHBA website (https://human.brain-map.org).

## Supplementary Information

Supplementary Information.
