# Supplementary material for: Connectivity adaptations in dopaminergic systems define the brain maturity of investors
Source: Sci Rep. 2021 Jun 3;11:11671. doi: 10.1038/s41598-021-91227-x (PMC8175592; doi:10.1038/s41598-021-91227-x)
Supplement: Supplementary file 1 — Supplementary Information. [file 41598_2021_91227_MOESM1_ESM.pdf]

SUPPLEMENTARY INFORMATION

Connectivity Adaptations in Dopaminergic Systems Define the Brain Maturity of Investors

Elena Ortiz-Teran[1,2*], Ibai Diez[1], Jorge Sepulcre[1], Joaquin Lopez-Pascual[3], Tomas Ortiz[4]

[1] Gordon Center for Medical Imaging, Department of Radiology, Massachusetts General Hospital, Harvard Medical School, Boston, Massachusetts 02115, USA; [2] Facultad de Ciencias Jurídicas y Sociales, Universidad Rey Juan Carlos, 28032 Madrid, Spain; [3] Departamento de Economía de la Empresa, Facultad de Ciencias Jurídicas y Sociales, Universidad Rey Juan Carlos, 28032 Madrid, Spain; [4] Departamento de Medicina Legal, Psiquiatría y Patología, Facultad de Medicina, Universidad Complutense de Madrid, 28040 Madrid, Spain.

*** Corresponding author:** ortizterane@gmail.com

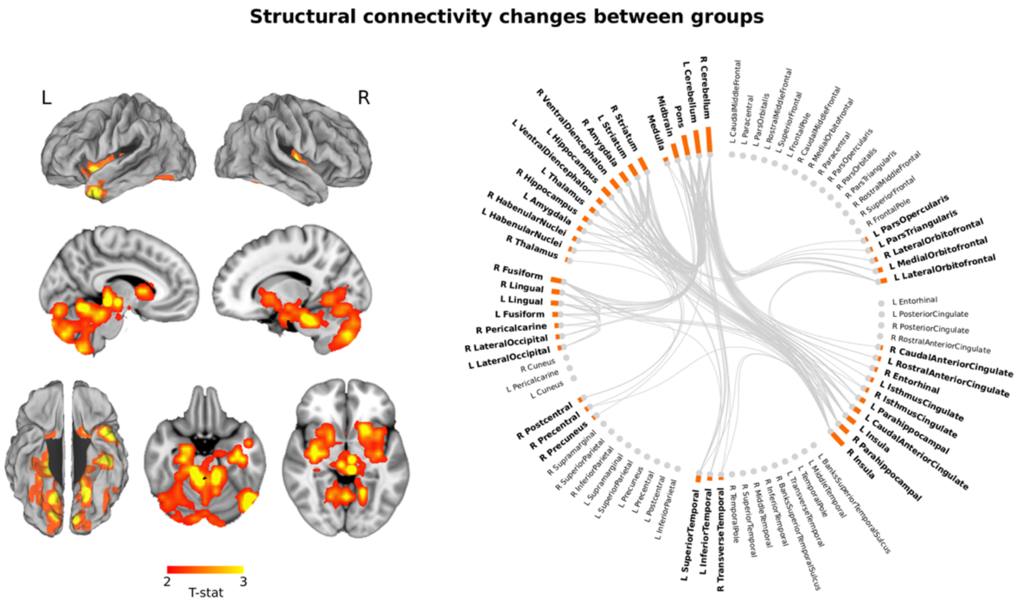

Figure S1: Connectogram of structural connectivity strength based on the weighted-degree of link-level interaction analysis maps between groups.

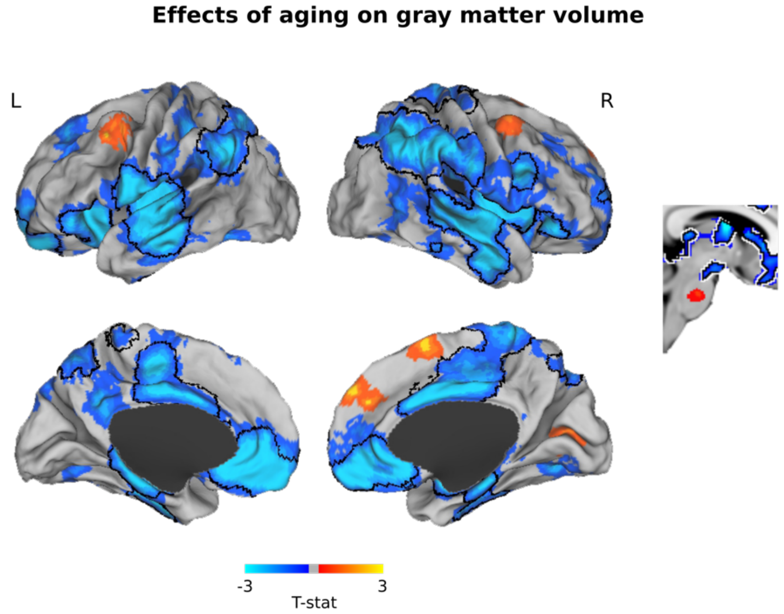

Figure S2: To control for the effect of age in our data we introduced the age as a cofounding factor in the statistical analysis. This figure shows the statistically significant effect of age in the data (lower volume in older subjects) that it was removed for all the analyses.

Table S1: Brain areas corrected for multiple comparisons, where senior investors exhibited higher gray matter volume, displayed in Figure 1A.

	HEMISPHERE	PEAK MNI COORDINATES	T-STAT
Frontal Pole	R	4, 56, -14	4.99
Frontal Medial Cortex	R	0, 54, -14	4.91
Angular Gyrus	R	52, -54, 44	4.29
Superior Parietal Lobule	R	34, -52, 52	4.16
Lateral Occipital Cortex	R	54, -60, 48	4.00
Thalamus	R	6, -28, 10	3.57
Inferior Temporal Gyrus	R	46, -26, -20	3.56
Temporal Fusiform Cortex	R	38, -22, -24	3.56
Putamen	R	34, 0, 4	3.43
Insular Cortex	R	34, 14, -8	3.28
Amygdala	R	32, -4, -20	3.13
Insular Cortex	L	-40, -14, -2	5.34
Medial Frontal Cortex	L	-10, 50, -14	4.26
Middle Temporal Gyrus	L	-66, -12, -10	4.19
Frontal Pole	L	-2, 64, -14	3.93
Putamen	L	-22, 20, -4	3.85
Thalamus	L	-20, -26, 12	3.54
Hippocampus	L	-26, -20, -18	3.40
Temporal Fusiform Cortex	L	-40, -22, -30	3.34
Superior Temporal Gyrus	L	-54, -12, -4	3.30
Frontal Orbital Cortex	L	-28, 22, -20	4.19
Caudate	L	-16, 20, -6	3.18
Accumbens	L	-14, 20, -8	3.03
Inferior Temporal Gyrus	L	-44, -16, -26	3.01
